# DOA Estimation on One-Bit Quantization Observations through Noise-Boosted Multiple Signal Classification

**DOI:** 10.3390/s24144719

**Published:** 2024-07-20

**Authors:** Yan Pan, Li Zhang, Liyan Xu, Fabing Duan

**Affiliations:** 1 College of Mathematics and Systems Science, Shandong University of Science and Technology, Qingdao 266590, China; qymzl5@163.com; 2 School of Electronic Information, Qingdao University, Qingdao 266071, China; xuliyan@qdu.edu.cn; 3 Institute of Complexity Science, Qingdao University, Qingdao 266071, China; fabingduan@qdu.edu.cn

**Keywords:** direction of arrival, one-bit quantization, noise-boosted quantizer unit, multiple-signal classification

## Abstract

Due to the low-complexity implementation, direction-of-arrival (DOA) estimation-based one-bit quantized data are of interest, but also, signal processing struggles to obtain the demanded estimation accuracy. In this study, we injected a number of noise components into the receiving data before the uniform linear array (ULA) composed of one-bit quantizers. Then, based on this designed noise-boosted quantizer unit (NBQU), we propose an efficient one-bit multiple signal classification (MUSIC) method for estimating the DOA. Benefiting from the injected noise, the numerical results show that the proposed NBQU-based MUSIC method outperforms existing one-bit MUSIC methods in terms of estimation accuracy and resolution. Furthermore, with the optimal root mean square (RMS) of the injected noise, the estimation accuracy of the proposed method for estimating DOA can approach that of the MUSIC method based on the complete analog data.

## 1. Introduction

Direction-of-arrival (DOA) estimation [1,2,3,4,5,6] has attracted considerable attention in the field of array signal processing, and its applications to wireless communications, radar, and sonar are of substantial practical significance. A number of DOA estimation algorithms have been continuously developed, particularly those based on uniform linear arrays (ULAs) [1,2] and sparse linear arrays (SLAs) [3,4,5,6]. Among these algorithms, the maximum likelihood and the subspace-based estimation methods, such as multiple-signal classification (MUSIC) [7], estimating signal parameters via rotational invariance techniques (ESPRIT) [8], and their variants [9,10], are particularly noteworthy due to their super-resolution capabilities. In addition, compressed sensing techniques [11,12], discrete Fourier transform methods [13,14], and deep learning approaches [15,16] have also been validated as effective strategies for DOA estimation.

However, the above-mentioned methods [7,8,9,10,11,12,13,14,15,16] usually assume ideal analog-to-digital converters and high-resolution quantization at the receiver. This assumption results in higher hardware costs and increased system power consumption, which bring substantial challenges for practical implementations. It is noted that the widespread use of low-power and low-complexity sensors in wireless networks and digital communication systems offers an answer to the demanding requirements of cost constraints. In particular, one-bit quantization DOA estimation, due to its low cost and low complexity in the hardware implementation, has gained considerable interest [17,18,19,20].

Many studies on one-bit quantization DOA estimation [21,22,23] are dedicated to improving the reconstruction of the unquantized covariance matrix from one-bit data by taking advantage of the arcsine law [24]. For instance, a one-bit MUSIC method is proposed in Ref. [25], wherein a one-bit covariance matrix can be approximated using a combination of a scaled unquantized covariance matrix and a scaled identity. Then, the combined one-bit covariance matrix is straightforwardly applied to compute the MUSIC spectrum, especially at a low signal-to-noise ratio (SNR). Moreover, the analytical performance bounds for the DOA estimation from one-bit quantized data were established in [21,26,27]. In Ref. [21], a closed-form expression of one-bit the Cramér-Rao bound (CRB) for a two-sensor array, which depends on the input SNR and the number of sensors or snapshots, was derived based on the quadrivariate orthant probability. In Ref. [27], a pessimistic approximation of CRB was obtained from one-bit sparse linear array data, and a new MUSIC-based DOA algorithm, which constructs an enhanced estimation of the normalized covariance matrix, is utilized. By exploiting the signal sparsity in the spatial domain, one-bit compressed sensing methods [28,29,30,31] have been addressed by modeling the DOA estimation problem as a sparse recovery one. Recently, the applications of quantization thresholds for the estimation of the full covariance matrix have been extensively studied. The implementation of non-zero deterministic thresholds [32] and the time-varying quantization thresholds [33,34] have been theoretically investigated to achieve precise and reliable covariance matrix estimates. In Ref. [35], the theoretical analysis of the threshold-based recovery scheme is proposed by computing the inverse of the Fisher information matrix.

It must be noted that, one-bit quantization, which only records the sign of real and imaginary parts of measurements, does lose some information of DOA estimation. This loss naturally leads to the degradation of performance in certain sub-optimal estimation algorithms when compared to the unquantized scenario. Therefore, two highly significant and meaningful challenges arise: how to improve the DOA estimation accuracy based on low-resolution sensor observations, such as one-bit data, and whether this estimation accuracy can reach or approach the accuracy achieved with unquantized data.

To address these critical questions, we deliberately injected noise into the data before one-bit quantizers, and then established a structure of a noise-boosted quantizer unit (NBQU) for enhancing the estimation accuracy of the DOA. This method primarily stems from the exploitation of the positive effects of noise, which have been extensively researched and employed in various nonlinear signal processing tasks [36,37,38,39,40]. For example, a quad-stable nonlinearity-based logical stochastic resonance was developed, which effectively extracts noise energy for enhancing logic information [36]. Additionally, combining noise injection into input weak signals and an adaptive multiparameter-adjusting Duffing system, gray images can be obtained using the data preprocessing technique for convolutional neural networks to obtain fault enhancement diagnoses [38]. With the assistance of noise, a mapping method converts the outputs of the Duffing system into gray images. In Ref. [40], an interesting detection scheme employing the matched stochastic resonance effect is established for weak-signal detection. It is observed [40] that an optimal noise intensity exists, which maximizes the output SNR of the designed system. For DOA estimation problems, a pseudo-noise resampling scheme [41,42,43] has been proposed for full-precision data, which is to generate estimator banks by combining underlying resampled estimators with the measured data perturbed by artificially generated pseudo-random noise. Through employing this strategy, a dilemma to be confronted is a trade-off between the computational cost and threshold performance. Inspired by noise benefits [36,37,38,39,40] and pseudo-noise resampling schemes [41,42,43], here, we designed the structure of an NBQU to improve the DOA estimation accuracy based on low-resolution sensor observations of one-bit data with noise injection. Subsequently, based on the outputs of the array of designed NBQUs, the MUSIC algorithm was implemented for the DOA estimation, named the NBQU-based MUSIC method. It has been shown that the injection of noise can smooth the transfer function of a one-bit quantizer, thereby improving the accuracy and resolution of DOA estimation. In comparison to the pseudo-noise resampling scheme, which implements multiple MUSIC estimations many times in numerical experiments, our proposed NBQU-based MUSIC algorithm executes only once in each numerical test and requires less computation time. Furthermore, experimental comparisons between the proposed NBQU-based MUSIC algorithm, the one-bit MUSIC methods in [21,25], and the traditional MUSIC algorithm based on the complete analog data were carried out. It is demonstrated that the proposed NBQU-based MUSIC method has a better estimation performance than the existing one-bit MUSIC methods. Furthermore, with the help of the optimally injected noise, the estimation accuracy of the proposed method for estimating DOA is very close to that of the MUSIC method based on the analog data.

## 2. One-Bit Observation Model

Consider a ULA comprising *M*-element sensors with an inter-element spacing *d*, assumed to be λ/2, where λ represents the signal wavelength, and suppose that *P* (P<M) far-field uncorrelated narrowband signals are incident on this ULA. The input $x\left(t\right)={\left[x_{1}\left(t\right),x_{2}\left(t\right),\cdots ,x_{M}\left(t\right)\right]}^{⊤}\in C^{M\times 1}$ of the array at time instant *t* can be expressed as

(1) x(t)=A(θ)s(t)+n(t),t=1,2,⋯,N,

where the vector $\theta ={\left[\theta_{1},\theta_{2},\cdots ,\theta_{P}\right]}^{⊤}\in R^{P\times 1}$ represents DOAs of the signals to be estimated, and $A\left(\theta \right)=[a\left(\theta_{1}\right),\cdots ,a\left(\theta_{P}\right)]$ is the steering matrix with its *p*-th column $a\left(\theta_{p}\right)={\left[1,e^{-j\frac{2\pi }{\lambda }d\sin\left(\theta_{p}\right)},\cdots ,e^{-j\frac{2\pi }{\lambda }\left(M-1\right)d\sin\left(\theta_{p}\right)}\right]}^{⊤}$. Here, $s\left(t\right)={\left[s_{1}\left(t\right),s_{2}\left(t\right),\cdots ,s_{P}\left(t\right)\right]}^{⊤}\in C^{P\times 1}$ is the random signal vector, and $n\left(t\right)={\left[n_{1}\left(t\right),n_{2}\left(t\right),\cdots ,n_{M}\left(t\right)\right]}^{⊤}\in C^{M\times 1}$ represents the additive sensor noise with power $\sigma_{n}^{2}$. The signal waveforms s(t) and the sensor noise n(t) are assumed to be uncorrelated, and both are modeled as independent, zero-mean, circular, complex Gaussian random processes. Here, ${\left(\cdot \right)}^{⊤}$ denotes the transpose operation.

The one-bit quantized samples can be obtained as

(2) $$y\left(t\right)=Q\left(x\left(t\right)\right)=\frac{1}{\sqrt{2}}\left[\mathrm{sign}\left(R\{x(t)\}\right)+j\cdot \mathrm{sign}\left(I\{x(t)\}\right)\right],$$

where the function sign(x) returns 1 for x≥0 and −1 otherwise. Here, R{·} and I{·} denote the real and imaginary parts of a complex number, respectively. The covariance matrix of the unquantized data x(t) is given by $R_{x}=E\left[x\left(t\right)x^{H}\left(t\right)\right]=AR_{s}A^{H}+\sigma_{n}^{2}I$, where $R_{s}=E\left[s\left(t\right)s^{H}\left(t\right)\right]$ is the covariance matrix of the signal waveforms s(t), and I represents the identity matrix. Here, E[·] and ${\left(\cdot \right)}^{H}$ denote the expectation operator and the Hermitian transpose. While, the covariance matrix $R_{y}=E\left[y\left(t\right)y^{H}\left(t\right)\right]$ corresponds to the quantized data y(t) of Equation (2).

It has been demonstrated [21,24,44] that, for one-bit DOA estimation from ULAs, the unquantized covariance matrix $R_{x}$ can be reconstructed from the covariance matrix $R_{y}$ based on the arcsine law. Subsequently, the one-bit MUSIC algorithm [25] can be straightforwardly utilized for establishing the resconstructed covariance matrix with a moderate performance loss. However, due to the nonlinearity of one-bit samples, the noise and signal subspaces may not always be completely separated by this straightforward approach, resulting in moderate degradation in DOA estimation accuracy. Therefore, the problem of DOA estimation from one-bit samples remains an interesting challenge that will be addressed as follows.

## 3. MUSIC Estimation Approach Based on Noise-Boosted Quantization

**Figure 1:**
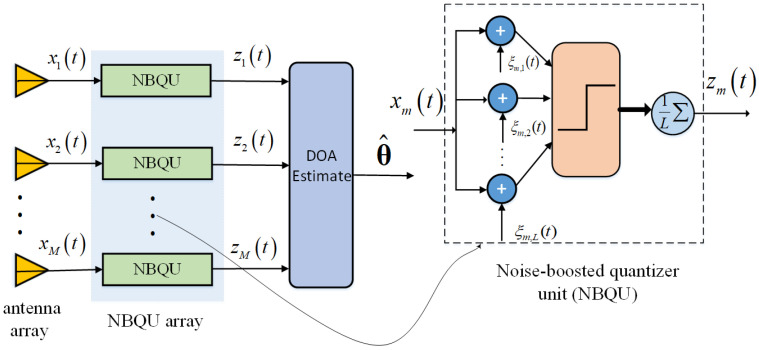
Block diagram of the array of noise-boosted quantizer units.

In order to improve the estimation accuracy of DOAs from one-bit quantized samples, we designed an array of NBQUs, as shown in Figure 1, where the received signal $x_{m}\left(t\right)$ at the *m*-th sensor node is artificially added with *L* groups of noise components $\xi^{m}\left(t\right)={\left[\xi_{m,1}\left(t\right),\cdots ,\xi_{m,L}\left(t\right)\right]}^{⊤}\in C^{L\times 1}$ and the one-bit quantized outputs are averaged as noise-modified measurements. Here, the injected noise components $\xi^{m}\left(t\right)$ are assumed to be mutually independent zero-mean, circular complex Gaussian process with the covariance matrix $\sigma_{\xi }^{2}I$, where $\sigma_{\xi }$ is the root mean square (RMS) of the injected noise. As illustrated in Figure 1, the average output of the *m*-th NBQU node can calculated as

(3) $$\begin{array}{ccc} z_{m}\left(t\right)\;\;\;\; & = & \;\;\;\;\frac{1}{L}\sum_{l=1}^{L}Q\left[x_{m}\left(t\right)+\xi_{m,l}\left(t\right)\right]\\ & = & \;\;\;\;\frac{1}{\sqrt{2}}\left\{\frac{1}{L}\sum_{l=1}^{L}\mathrm{sign}\left[R\left(x_{m}\left(t\right)+\xi_{m,l}\left(t\right)\right)\right]\right\}+\frac{j}{\sqrt{2}}\left\{\frac{1}{L}\sum_{l=1}^{L}\mathrm{sign}\left[I\left(x_{m}\left(t\right)+\xi_{m,l}\left(t\right)\right)\right]\right\}\\ & = & \;\;\;\;\frac{1}{\sqrt{2}}g\left(R\left(x_{m}\left(t\right)+\xi_{m,l}\left(t\right)\right)\right)+\frac{j}{\sqrt{2}}g\left(I\left(x_{m}\left(t\right)+\xi_{m,l}\left(t\right)\right)\right)\\ & = & \;\;\;\;\tilde{Q}\left[x_{m}\left(t\right)\right], \end{array}$$

where $\tilde{Q}\left(\cdot \right)=\left[g\left(R\left(\cdot \right)\right)+jg\left(I\left(\cdot \right)\right)\right]/\sqrt{2}$, and the transfer function of the designed NBQU is given by

(4) $$g\left(x;L,\sigma_{\xi }\right)=\frac{1}{L}\sum_{l=1}^{L}\mathrm{sign}\left[x+\frac{\sigma_{\xi }}{\sqrt{2}}{\bar{\xi }}_{l}\right].$$

**Figure 2:**
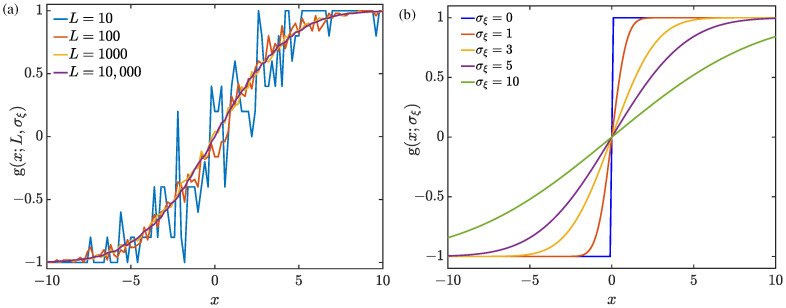
Input–output characteristics of the NBQU transfer function (**a**) $g(x;L,\sigma_{\xi })$ in Equation (4) with a given noise RMS $\sigma_{\xi }=5$ for different group numbers *L* and (**b**) $g(x;\sigma_{\xi })$ in Equation (5) with different values of the noise RMS $\sigma_{\xi }$.

Here, ${\bar{\xi }}_{l}$ represents the standardized random variable with zero mean and unit variance. It is seen in Equation (4) that, for a given *x*, the transfer function g(x) takes discrete values within a bounded interval [−1,1], and the number of discrete values for g(x) also increases as the group size *L* increases. For a certain noise RMS $\sigma_{\xi }$, Figure 2a shows the input–output characteristic of the transfer function g(x) for different group numbers *L* of added noise. It can be seen in Figure 2a that, as *L* becomes sufficiently large, the input–output characteristic of g(x) gradually evolves into a continuous and smooth function.

In the limit case of *L* approaching infinity, the transfer function g(x) in Equation (4) of the designed NBQU-array can be approximated as

(5) $$\begin{array}{ccc} g(x;\sigma_{\xi }) & = & \lim_{L\to \infty }\frac{1}{L}\sum_{l=1}^{L}\mathrm{sign}\left[x+\frac{\sigma_{\xi }}{\sqrt{2}}{\bar{\xi }}_{l}\right]\\ & = & E_{\bar{\xi }}\left\{\mathrm{sign}\left[x+\frac{\sigma_{\xi }}{\sqrt{2}}{\bar{\xi }}_{l}\right]\right\}\\ & = & 2\Phi \left(\frac{x}{\sigma_{\xi }/\sqrt{2}}\right)-1\\ & = & \mathrm{erf}\left(\frac{x}{\sigma_{\xi }}\right) \end{array}$$

with a tunable parameter $\sigma_{\xi }$. Here, $\Phi \left(x\right)=\frac{1}{2}+\frac{1}{2}\mathrm{erf}\left(x/\sqrt{2}\right)$ denotes the cumulative distribution function of a standard Gaussian random variable, $\mathrm{erf}\left(x\right)=2\int_{0}^{x}\exp\left(-u^{2}/2\right)du/\sqrt{\pi }$ is the error function, and $E_{\bar{\xi }}\left[\cdot \right]$ indicates the expectation operator with respect to the standard Gaussian distribution.

It is interesting to note that, for a small input *x*, the first-order Taylor expansion to the function g(x) can be expressed as

(6) $$g\left(x;\sigma_{\xi }\right)=\mathrm{erf}\left(\frac{x}{\sigma_{\xi }}\right)\approx \frac{2}{\sqrt{\pi }}\frac{1}{\sigma_{\xi }}x+O\left(x^{3}\right),$$

which indicates that the transfer function $g(x;\sigma_{\xi })$ is almost linear for small inputs. It is shown in Figure 2b that, with the assistance of the injected noise, the transfer function $g(x;\sigma_{\xi })$ of the quantizers is smoothed as a bounded and derivable continuous function. Therefore, the received signals can be utilized to implement the DOA estimation by optimally adjusting the noise RMS $\sigma_{\xi }$.

For the output vector $z\left(t\right)={\left[z_{1}\left(t\right),z_{2}\left(t\right),\cdots ,z_{M}\left(t\right)\right]}^{⊤}$ of the designed NBQU array, the covariance matrix of z(t) is defined as $R_{z}=E\left[z\left(t\right)z^{H}\left(t\right)\right]$, which is a nonlinear function of the RMS $\sigma_{\xi }$. Therefore, some subspace-based methods involving the eigen decomposition of the covariance matrix can be optimized by adjusting the RMS of the added noise.

In practical scenarios, the covariance matrix $R_{z}$ is usually estimated by the sample covariance matrix

(7) $${\hat{R}}_{z}=\frac{1}{N}\sum_{t=1}^{N}z\left(t\right)z^{H}\left(t\right).$$

Then, the sample covariance matrix ${\hat{R}}_{z}$ can be decomposed into signal and noise subspaces according to its eigenvalues, i.e.,

(8) $${\hat{R}}_{z}={\hat{U}}_{s}{\hat{\Lambda }}_{s}{\hat{U}}_{s}^{H}+{\hat{U}}_{n}{\hat{\Lambda }}_{n}{\hat{U}}_{n}^{H}.$$

Based on the eigen decomposition of ${\hat{R}}_{z}$ in Equation (8), the MUSIC method [7,45] can be implemented for obtaining the higher resolution subspace-based DOA estimation. Here, ${\hat{U}}_{s}$ and ${\hat{U}}_{n}$ indicate the estimates of the signal and noise subspaces, and  ${\hat{\Lambda }}_{s}$ and ${\hat{\Lambda }}_{n}$ correspond to the diagonal matrices of the estimated *P* largest eigenvalues and the M−P smallest eigenvalues, respectively. According to the orthogonality between the signal subspace and the noise subspace, the DOAs of the sources are estimated from the peaks of the spectrum

(9) $$P_{\mathrm{MUSIC}}\left(\theta \right)=P_{\mathrm{MUSIC}}\left(\theta ;\sigma_{\xi }\right)=\frac{1}{a^{H}\left(\theta \right){\hat{U}}_{n}{\hat{U}}_{n}^{H}a\left(\theta \right)}.$$

Thus, the *P* values of θ that correspond to the peaks of MUSIC space spectrum $P_{\mathrm{MUSIC}}\left(\theta \right)$ are obtained as the estimates ${\hat{\theta }}_{p}$ of signal DOAs for p=1,2,⋯,P. A summarized description of the NBQU-based MUSIC algorithm for the designed array is presented in Algorithm 1.

**Figure 3:**
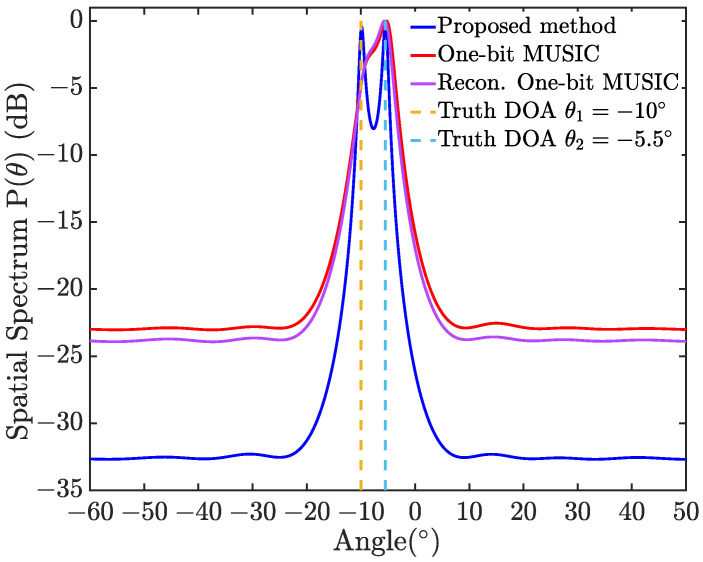
MUSIC spectrums for two sources with $\theta_{1}=-10^{\circ }$ and $\theta_{2}=-5.5^{\circ }$ obtained through the NBQU-based MUSIC in Algorithm 1, the one-bit MUSIC in [25], and the Recon. One-bit MUSIC in [21,25], respectively. Here, L=1000 groups of injected noise samples with the RMS $\sigma_{\xi }=6$ were employed.

For instance, Figure 3 depicts the spectrums $P_{\mathrm{MUSIC}}\left(\theta \right)$ calculated by the proposed NBQU-based MUSIC algorithm, the one-bit MUSIC in [25], and the reconstruction one-bit MUSIC (Recon. One-bit MUSIC) in [21,25], respectively, with the SNR being $10\log_{10}\left(\sigma_{s}^{2}/\sigma_{\xi }^{2}\right)=10$ dB. It is shown in Figure 3 that the proposed Algorithm 1 can resolve the sources for two closely spaced DOAs of $\theta_{1}=-10^{\circ }$ and $\theta_{2}=-5.5^{\circ }$, while the one-bit MUSIC in [25] and the Recon. One-bit MUSIC in [21,25] fail. The optimal RMS $\sigma_{\xi }=6$ of the injected noise aiming to minimize the estimation error can be obtained via grid search.
**Algorithm** **1** NBQU-based MUSIC**Input:**  Array receiving measurements x(1),x(2),⋯,x(N), the number of signal sources *P*.**Output:**  the DOA estimate ${\hat{\theta }}_{p}$, p=1,2,⋯,P.  1:Generate *L* groups of M×1 pseudo-noise $\xi_{1}\left(t\right),\xi_{2}\left(t\right),\cdots ,\xi_{L}\left(t\right)$ with a certain RMS $\sigma_{\xi }$ and $\xi_{l}\left(t\right)={\left[\xi_{1,l},\xi_{2,l},\cdots ,\xi_{M,l}\right]}^{⊤}$;  2:Perform one-bit quantization sampling on the noise-modified data $x\left(t\right)+\xi_{l}\left(t\right)$ for l=1,2,⋯,L, and average them at the fusion center to obtain NBQU samples z(1),z(2),⋯,z(N);  3:Construct the sample covariance matrix ${\hat{R}}_{z}=\frac{1}{N}\sum_{t=1}^{N}z\left(t\right)z^{H}\left(t\right)$ based on z(t);  4:Perform the eigen decomposition of ${\hat{R}}_{z}$ in Equation (8) and obtain the estimate of noise subspace ${\hat{U}}_{n}$;  5:Calculate the spectrum $P_{\mathrm{MUSIC}}\left(\theta \right)$ of Equation (9);  6:Output the DOA estimates according to the locations of the *P* highest peaks of the spectrum.

The computational complexity of Algorithm 1 is analyzed in Table 1 for each step, where *C* denotes the number of grid points. Due to the generation of *L* groups of noise components, the proposed algorithm requires approximately LMN additions, which is *L* times the number of additions needed by the traditional one-bit MUSIC algorithm in steps 1 and 2. However, considering the improvement in estimation accuracy, the increase in the number of injected noise groups *L* represents a reasonable trade-off.

## 4. Main Results

**Figure 4:**
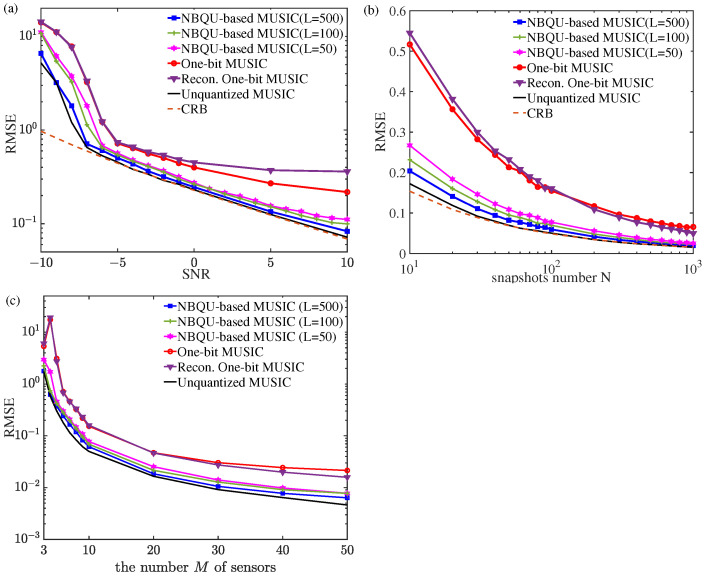
RMSEs of DOA estimation versus (**a**) the input SNR (N=50 snapshots), (**b**) the snapshot number *N* at an input SNR of 10 dB, and (**c**) the sensor number *M* at an input SNR of 10 dB and snapshots N=100.

Simulation experiments were performed to validate the effectiveness of the proposed NBQU-based MUSIC algorithm in terms of the root mean square error (RMSE) defined as

(10) $$\mathrm{RMSE}=\sqrt{\frac{1}{RK}\sum_{r=1}^{R}\sum_{k=1}^{P}{\left({\hat{\theta }}_{k,r}-\theta_{k}\right)}^{2}}$$

where *R* is the number of Monte Carlo trails, *P* is the number of sources, ${\hat{\theta }}_{k,r}$ denotes the DOA estimation result of the *k*-th source at the *r*-th Monte Carlo trail, and $\theta_{k}$ is the true DOA value of the *k*-th source. For instance, we observed a ULA with M=10 sensors, and two uncorrelated equal-power Gaussian sources (P=2) were with DOAs $\theta_{1}=-10^{\circ }$ and $\theta_{2}=3.5^{\circ }$. The RMSs in Equation (10) of the three considered estimation methods, i.e., the proposed NBQU-based MUSIC Algorithm 1, the one-bit MUSIC from [25], and the Recon. One-bit MUSIC from [21,25], are shown in Figure 4, where the background noise power takes $\sigma_{n}^{2}=1$, and the input SNR changes with the signal power $\sigma_{s}^{2}$ without loss of generality. Each point was obtained through $10^{3}$ Monte-Carlo trails.

**Figure 5:**
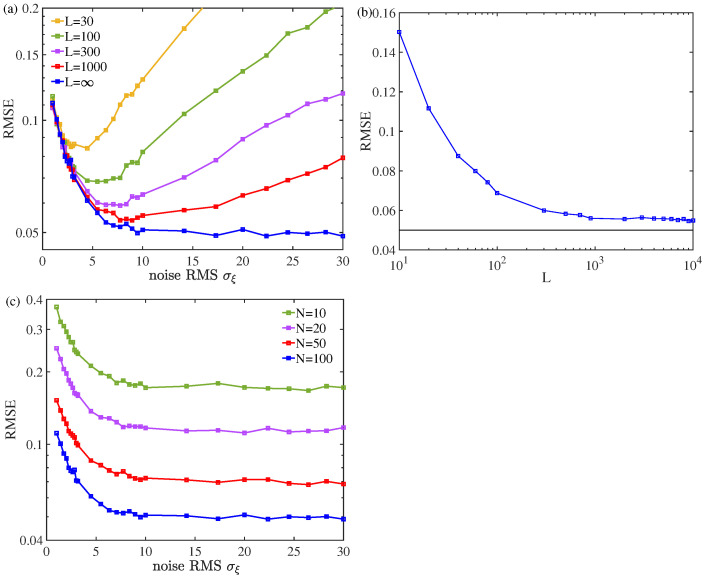
RMSEs of DOA estimation versus (**a**) the noise RMS $\sigma_{\xi }$ for different group numbers *L* (snapshot number N=100), (**b**) the group number *L* of injected noise components (noise RMS $\sigma_{\xi }=6$ and snapshot number N=100), and (**c**) the snapshot numbers *N* (L→∞ and $\sigma_{\xi }=6$). Here, the input SNR was fixed at 10 dB.

Figure 4a illustrates the RMSEs of Equation (10) versus the input SNR for M=10 sensors and N=50 snapshots. The results of the proposed NBQU-based method for noise groups L=500, L=100, and L=50 were obtained by injecting noise with RMS values $\sigma_{\xi }=6$, $\sigma_{\xi }=4$, and $\sigma_{\xi }=3$, respectively. It can be observed from Figure 4a that, as the noise group number of *L* increases, the RMSE of the proposed estimation method decreases. These results are also consistent with Figure 5b. Compared to the one-bit MUSIC and the Recon. One-bit MUSIC methods, the proposed NBQU-based MUSIC algorithm performs better in terms of the RMSEs of Equation (10). Moreover, the RMSE of the NBQU-baded method for L=500 (squares) achieves a performance very close to that of the unquantized case. In particular, under the conditions of low SNRs, the proposed method exhibits a significantly superior performance compared to the other two estimation methods.

Furthermore, the unquantized estimation results and CRB [46] were also given as a benchmark. It is noted in Figure 4a–c that the performance difference between the one-bit MUSIC and the Recon. One-bit MUSIC is slight, and this result also agrees with the conclusion in Ref. [25]. Under the condition of the input SNR of 10 dB, Figure 4b illustrates the variation in the RMSEs of the three considered methods versus the number *N* of snapshots. As the number *N* varies, the proposed NBQU-based MUSIC method for L=500 still obtains a lower RMSE very close to that of the unquantized case; however, the one-bit MUSIC and the Recon. One-bit MUSIC methods have larger RMSEs in the DOA estimation. The RMSEs of the proposed NBQU-baded method are also provided under different numbers of sensors in the ULA, as shown in Figure 4c. The results show that the RMSEs of the proposed NBQU-based method, the one-bit MUSIC method, and the Recon. One-bit MUSIC method all decrease as the number of sensors increases. However, the RMSE curves of the NBQU-based methods are consistently lower than those of the other two methods. This finding demonstrates the significant value and practical applications of the proposed noise-boosted estimation method.

Aiming to exploit the noise benefits in detail, Figure 5a depicts the RMSEs of DOA estimation versus the injected noise RMS $\sigma_{\xi }$ for different group numbers *L* of injected noise components and the snapshot number *N*, respectively. For a given finite group number *L*, it is seen in Figure 5a that there is an optimal value of noise RMS $\sigma_{\xi }$ corresponding to the minimum of the RMSE of DOA estimation. Of course, for a given number N=100 and at a fixed noise RMS $\sigma_{\xi }=6$, the RMSEs of DOA estimation decrease and gradually approach to those of the unquantized case, as shown in Figure 5b. In addition, for a given snapshot number *N*, it is illustrated in Figure 5c that the injected noise ξ(t) exhibits a quite stable improvement of the RMSEs of DOA estimation for a wide range of noise RMS $\sigma_{\xi }$.

**Figure 6:**
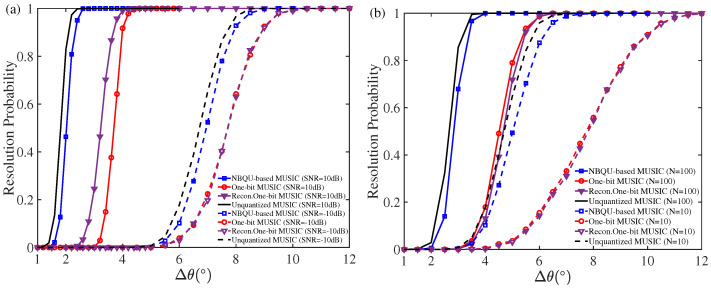
Resolution probability versus angular separation (**a**) in SNRs 10 dB and −10 dB with N=500 snapshots; (**b**) for N=10 and N=100 snapshots in input SNR of 10 dB. Here, L=500 groups of the injected noise with RMS $\sigma_{\xi }=6$.

Furthermore, experiments on the resolution ability for evaluating two closely-spaced targets were carried out. Here, we considered two uncorrelated targets of $\theta_{1}=-10^{\circ }$ and $\theta_{2}=-10+\Delta \theta$, where Δθ uniformly takes values in $\left[1^{\circ },12^{\circ }\right]$ by the interval $1^{\circ }$. When the angle estimates of ${\hat{\theta }}_{p}$ for p=1,2 satisfy $|{\hat{\theta }}_{1}-\theta_{1}\left|<\right|\theta_{1}-\theta_{2}|/2$ and $|{\hat{\theta }}_{2}-\theta_{2}\left|<\right|\theta_{1}-\theta_{2}|/2$, the two signals are assumed to be resolved. Figure 6 plots the resolution probability, being the percentage of successful resolution times among the total Monte Carlo simulations, versus the angular separation Δθ for different estimation algorithms. Here, the points in Figure 6 were obtained from $10^{3}$ Monte Carlo trials. It is noticed in Figure 6 that the resolution ability of the proposed NBQU-based MUSIC method achieves a rather comparable performance in comparison to the MUSIC method based on the complete analog data. In particular, for the low-SNR region and the small snapshot number, the proposed method greatly exceeds that of the other considered MUSIC algorithms, as shown in Figure 6.

## 5. Conclusions

In this study, we intentionally injected a number of mutually independent noise components into the measurements before the one-bit quantizer and designed a noise-boosted ULA for the DOA estimation. Then, based on the designed array, we proposed the NBQU-based MUSIC algorithm and compared it with the one-bit MUSIC and the Recon. One-bit MUSIC methods. It is demonstrated that, with the help of the injected noise, the proposed DOA estimation algorithm exhibits a significant improvement over the existing one-bit MUSIC methods in terms of estimation accuracy and resolution ability. Furthermore, it can approach the estimation performance of MUSIC algorithm based on unquantized measurements for a sufficiently large group number *L*. Additionally, this noise-boosted ULA for DOA estimation is also applicable to other DOA estimation algorithms, including ESPRIT, root-MUSIC, root-weighted subspace fitting, and others. The enhancement provided by noise injection holds true across these estimation methods, though the experimental results are not shown here for simplicity.

It is noted that, in order to obtain the comparable estimation accuracy achieved in unquantized cases, the proposed NBQU-based MUSIC algorithm requires a large number of injected noise sequences, specifically $L=10^{3}$. This may not be applicable in practical applications. In order to reduce the number *L*, an optimal weight vector $w_{m}={\left(Y_{m}^{H}Y_{m}\right)}^{-1}Y_{m}^{H}x_{m}$ based on the least squares criterion [46], instead of the same fixed weight 1/L applied in Equation (3) can be introduced to the m-th array element in the designed ULA. With the optimal weight vector $w_{m}$, the experimental result shows that, under the condition of the input SNR of 10 dB and the number of snapshots N=50, the estimation RMSE of 0.083 achieved by injecting L=500 noise sequences (see Figure 4a) can be obtained utilizing only L=35 noise sequences. In such a case, the number *L* is greatly reduced, and the efficacy of the weight-based one-bit MUSIC method is worth further study. It will be highly significant to carry out real-world field experiments and improve our proposed algorithm for signal estimation tasks. These field experiments could involve the construction of microphone arrays to localize the direction of single- or multiple-sound sources, which will be implemented in our upcoming research.

There are still several open questions that require further investigation. For instance, how to determine the optimal noise RMS remains is a crucial task. Although the grid search is a fundamental approach, it exhaustively explores a predefined search space. More effective optimization algorithms, such as a genetic algorithm or particle swarm optimization, need to be explored and tested for searching for the optimal noise RMS. Here, we assumed that the injected noise follows a Gaussian distribution. Thus, it is worth considering the injection of other noise types and finding the optimal injected noise type to achieve the minimum RMSE of DOA estimation. This open question is also of significant interest. Specifically, exploring uniform quantizer noise [47], generalized Gaussian noise [48], and near-optimal noise models [37], with probability density functions that are parameterized by various kernel functions, may provide valuable insights for DOA estimation. Finally, the feasibility of the designed noise-boosted DOA estimator in a sparse array or a under non-Gaussian noisy environment, such as non-Gasussian impulsive noise [40] and Lévy noise [39], is still an open question for future study.

## Figures and Tables

**Table 1:** Computational complexity of the NBQU-based algorithm.

Step Order	Operation	Computational Complexity
1–2	constructing the noise-boosted measurements [z(1),⋯,z(N)]	O(LMN)
3	estimating the covariance matrix ${\hat{R}}_{z}$	$O(M^{2}N)$
4–6	applying the MUSIC algorithm to ${\hat{R}}_{z}$ to estimate DOAs	$O(M^{3}+M^{2}C)$

## Data Availability

Data are available on request from the corresponding author.
